# Modulation of TLR 3, 7 and 8 Expressions in HCV Genotype 3 Infected Individuals: Potential Correlations of Pathogenesis and Spontaneous Clearance

**DOI:** 10.1155/2014/491064

**Published:** 2014-07-13

**Authors:** Rushna Firdaus, Aritra Biswas, Kallol Saha, Anirban Mukherjee, Falguni Pal, Sujit Chaudhuri, Alok Chandra, Asokananda Konar, Provash Chandra Sadhukhan

**Affiliations:** ^1^I.C.M.R. Virus Unit Kolkata, ID and BG Hospital Campus, GB-4 (East Wing), 1st Floor, 57 Dr. Suresh Chandra Banerjee Road, Beliaghata, Kolkata, West Bengal 700010, India; ^2^Department of Gastroenterology, AMRI Hospitals Salt Lake, JC - 16 & 17, Salt Lake City, Kolkata 700091, India; ^3^Department of Medicine and Gastroenterology, Eastern Command Hospital, Alipore road, Kolkata 700027, India; ^4^Department of Gastroenterology, Peerless Hospital and B.K. Roy Research Centre, Kolkata 700094, India

## Abstract

*Background*. Hepatitis C virus is the major cause of chronic hepatitis worldwide which finally leads to the development of hepatocellular carcinoma. Toll like receptors (TLRs) play an important role in the course of many viral infections, but the role of TLRs in HCV pathogenesis has not been well elucidated so far. *Objective*. The aim of this study was to analyse the mRNA expression of TLRs 3, 7, and 8 in different stages of HCV infection including chronic, cirrhosis, interferon treated resolved, and relapsed cases. *Methodology*. Total RNA from whole blood was extracted and mRNA expression of TLRs 3, 7, and 8 genes was analyzed by quantitative real-time RT-PCR using *β*-Actin gene as an internal control. *Results*. This study consisted of 100 HCV infected individuals and twenty healthy controls. TLR 3 expression was found to be significantly elevated in individuals who had spontaneously cleared the virus (*p* < 0.001), whereas TLR 7 was found to be 3.26 times more elevated in patients with cirrhosis of liver. In IFN induced individuals, TLR 8 expression levels were found to be 2.28-fold elevated as compared to control population. *Conclusion*. TLRs 3, 7, and 8 are prime biomarker candidates for HCV infection mRNA expression analysis which might improve current therapeutic approaches.

## 1. Introduction

Hepatitis C virus (HCV) infection is an evolving public health problem globally and the major cause of chronic hepatitis worldwide which finally leads to the development of hepatocellular carcinoma (HCC) [1]. This virus infects 3% of world population and 180 million people are at risk for developing HCV related chronic liver disease [2]. The prevalence rate of HCV infection varies considerably from country to country [3]. In India, 20 million people are infected with HCV, according to study conducted by World Gastroenterology Organization [4].

HCV is an enveloped, single-stranded RNA virus which is prone to a lot of mutations which leads to constant alteration of its phenotype and formation of new quasispecies [5]. Due to genetic heterogeneity, this virus evades the immune system and establishes chronic infection within the patient's body [5]. The immune system can be broadly categorized into adaptive and innate immunity. In recent years, it has become clear that not only the adaptive but also the innate immunity system is involved in viral clearance [6]. The innate immune system represents the initial line of host defence mechanism against invading pathogen. Germ line encoded pattern recognition receptors (PRRs) recognize conserved molecules associated with microbial pathogens, known as microbe-associated molecular patterns (MAMPs) [6]. Toll like receptors (TLRs) are a class of PRRs that detect an increasingly broad range of pathogens and play an important role in host innate immunity [7].

Although majority of the work on TLR has focused on detection of bacteria and fungi, it is becoming increasingly apparent that viruses are also subject to innate sensing by TLRs [8]. Immune cells sense viral infection by detecting viral protein and/or nucleic acid, the double-stranded RNA (dsRNA), which is a byproduct of the replicative cycle of many ssRNA viruses [9]. Viral proteins and dsRNA are recognized through receptors such as evolutionary conserved TLR. Yet 10 human and 12 murine TLRs have been reported [9]; among them, TLR 3 has been identified to respond to dsRNA [10]. Evidences also suggested that single-stranded RNA stimulate TLRs 7 and 8, while unmethylated CpG-containing DNA have been identified to stimulate TLR 9 [11].

Immune responses, including cell-mediated immunity and type 1 IFNs, are vital in controlling and clearing HCV infection [12]. HCV evades the host immune system to sustain a chronic infection. Toll like receptors (TLRs) have been shown to play an important role in the course of many viral infections, but the role of TLRs in HCV pathogenesis has not been elucidated so far [11–14]. For instance, it is still unclear how the HCV mediated pathogenesis is involved in the HCV inactivation and alteration of functions of B cells and hepatocytes during acute and chronic HCV infection. Different structural parts of the HCV can be detected by the host innate immune system, which leads to cytokines secretion and induction of an antiviral state as well as inflammation processes.

TLR 3 is expressed on endosomal membranes (and the plasma membranes of some cells) and thus senses double-stranded dsRNA of viral origin and is expressed preferentially in dendritic cells. Once engaged, TLR 3 triggers the activation of interferon-regulatory factor-3 (IRF-3), a transcription factor playing a critical role in the induction of type I interferon and NF-*κ*B through signalling processes that require the protein TRIF. Type I IFN further upregulates TLR 3 in an autocrine/paracrine manner, a phenomenon linked to its antiviral gene defense action [15].

TLR 7 is sensor for viral single-stranded RNA (ssRNA) and appears to be preferentially expressed by plasmacytoid dendritic cells and B lymphocytes. This TLR also triggers IRF-7 mediated type I IFN, but, unlike TLR 3, the induction of IFN by TLR 7 is coupled to the adaptor protein myeloid differentiation primary response gene (MyD88) and not to TIR-domain-containing adapter-inducing interferon-*β* (TRIF). TLR 8 is phylogenetically related to TLR 7 and is also a sensor for ssRNA. It activates MyD88 and then activates NF-*κ*B which in turn further activates cytokines and chemokines to generate an immune response. HCV interferes with signals of the TLR 7 as NS5A interacts with MyD88 through the IFN sensitivity determining region (ISDR). The current treatment regimen against HCV is administering doses of single IFN-*α* subtype (2a or 2b), but TLR activation induces a host of interferon subtype responses; therefore, TLR agonists can help in improving efficacy of the antiviral therapy [16].

In the Indian context, there is limited evidence of the TLR and how it regulates the pattern of innate immune response among HCV genotype-3 infected patients, the major circulating genotype in this country. Thus, our study aimed to determine the relative mRNA expression levels of TLRs 3, 7, and 8 in patients who are HCV infected but treatment naive, patients who are undergoing IFN therapy, and patients who have spontaneously cleared the infection and in HCV patients who have progressed to cirrhosis of the liver. Understanding the variation of the different TLRs on the various stages of HCV infection may provide a new set of molecular markers for the progression of HCV infection and suggest additional antiviral targets.

## 2. Material and Methods

### 2.1. Ethics Statement

This work was a part of the study approved by the Institutional Ethical Committee, National Institute of Cholera and Enteric Diseases, Indian Council of Medical Research, Kolkata. Written as well as informed consent was obtained from all the study participants.

### 2.2. Study Subjects and Patient Criteria

A total of 100 HCV infected individuals were enrolled in this study as well as 20 healthy individuals (controls). Individuals enrolled in this study were treatment-naïve patients chronically infected with HCV (HCV patients, *n*= 20), patients who have spontaneously cleared HCV infection (*n*= 20), patients who have cleared the infection after interferon treatment: interferon induced HCV clearance (*n*= 20), HCV infected individuals with relapsed infection after completion of interferon treatment: relapsed cases (*n*= 20), and individuals with HCV related cirrhosis of liver (*n*= 20). This study group is comprised of chronic HCV infections only.

Hepatitis C is a long-term illness that occurs when the HCV remains in a person's body. When the infection persists for more than 6 months, it is termed as chronic infection. Over time, it can lead to serious liver problems, including liver damage, cirrhosis, liver failure, or liver cancer. Relapsed is defined as person who has achieved an undetectable level of virus during a prior treatment course of PEG/RBV and relapsed after treatment was stopped [17].

### 2.3. Biochemical Parameters

Liver function parameters like alanine aminotransferase (ALT) and aspartate aminotransferase (AST) were measured by kinetic rate methods (Beckman Coulter Synchron CX5Pro, USA).

In the reaction, ALT catalyzes the reversible transamination of L-alanine and *α*-ketoglutarate to pyruvate and L-glutamate. The pyruvate is then reduced to lactate in the presence of lactate dehydrogenase (LDH) with the concurrent oxidation of NADH to NAD. The system monitors the rate of change in absorbance at 340 nm over a fixed-time interval. The rate of change in absorbance is directly proportional to the ALT activity in the sample.

In the AST reaction, aspartate and *α*-ketoglutarate are first converted to glutamate and oxaloacetate which are converted by malate dehydrogenase to make malate and NAD. The conversion of the NADH chromophore to NAD^+^ product is measured at 340 nm and is proportional to the level of AST enzyme in the sample.

## 3. Viral RNA Detection and Quantitation

### 3.1. Extraction of HCV RNA from Serum

Five milliliters of whole blood was drawn from each patient by venipuncture; blood specimens used for the preparation of serum were collected in a serum separator tube with clot activator and gel. Viral RNA was extracted from serum samples. Briefly, 140 *μ*L of serum was used to isolate total viral RNA using QIAamp viral RNA Mini Kit (Qiagen, Hilden, Germany) according to the manufacturer's protocol and eluted in 50 *μ*L with elution buffer. The RNA was aliquoted and stored at −80°C for further usage.

### 3.2. Qualitative Detection of HCV RNA by Nested RT-PCR

Detection of HCV viral RNA was done by nested RT-PCR based on 5′noncoding region (5′NCR) of HCV genome described elsewhere [18]. Briefly, first round RT-PCR was done in 20 *μ*L total reaction volume containing 2 *μ*L isolated viral RNA and second round nested PCR was performed in 25 *μ*L total volume with 2 *μ*L of the first round PCR product. A band at 256 bp in 1.5% agarose gel stained with ethidium bromide was observed in gel documentation system (BioRad, USA) for HCV RNA positive samples.

### 3.3. Quantitation of HCV RNA in Serum

Quantitative HCV RNA was estimated in-house using ABI real-time RT-PCR kit (AgPath-ID One Step RT-PCR kit). The HCV primers and probe sequences were directed against the 5′NCR (noncoding region) of the HCV genome [18]. The primers and probe were designed in-house. The primer sequence H5UF: 5′ CCCCTGTGAGGACTWCTGTCTTC-3′, H5UR: GCAGACCACTATGGCTCTCC-3′, and probe sequence: 6 FAM-CTAGCCATGGCGTTAGTAYGAGTGTCG-MGB. HCV standards were the 4th WHO International Standard for HCV, NIBSC code 06/102. The viral loads in serum were expressed as international units per millilitre (IU/mL).

### 3.4. DNA Sequencing and Genotyping

Nested RT-PCR amplified amplicons of partial HCV core gene (405 bp) were gel purified and directly used for DNA sequencing in an automated DNA sequencer, model 3130XL (Applied Biosystems, Foster City, USA) using Big Dye terminator 3.1 kit (Applied Biosystems, Foster City, USA) [18]. The genotypes of the sequences obtained were determined using the NCBI genotyping tool [19].

## 4. Toll Like Receptor (TLR) Analysis

### 4.1. Extraction of Total RNA from Whole Blood

200 *μ*L of whole blood was used for the total cellular RNA extraction by using whole blood RNA purification Mini Kit (Thermo Fisher, Lithuania, EU) according to the manufacturer's protocol. Total RNA was eluted in 50 *μ*L buffer and kept at −80°C until further use. All samples were coprocessed to eliminate technical variations. RNA was quantified using a nanodrop BioPhotometer plus (Eppendorf, Germany) and equal amounts of RNA from controls and HCV infected patients were analyzed.

### 4.2. cDNA Synthesis and TLR Expression by Real-Time PCR

Reverse transcription of 1 *μ*g of total RNA into cDNA was performed using reverse transcription system of first-strand cDNA synthesis system (Thermo fisher, EU). The expression of TLR mRNA was investigated by quantitative real-time PCR in whole blood. The PCR primers used for TLR expression are described in Table 2. The reaction mixture contained 12.5 *μ*L Power SYBR Green PCR Master-mix plus with 2X SYBR Green dye (Applied Biosystems, USA); 0.5 *μ*mole of forward and reverse primers and 1 *μ*L of cDNA were used in a total reaction volume of 25 *μ*L. All amplifications and detections were carried out in ABI 7500 (Applied Biosystems, Foster City, USA) real-time PCR machine. At each cycle, accumulation of PCR products was detected by monitoring the increase in fluorescence by dsDNA-binding SYBR Green. After the PCR was performed, a dissociation/melting curve was constructed in the range of 55°C to 95°C. Data were analyzed using the ABI software. As a first step in data analysis, *β*-Actin was used as an internal control for normalization of all experiments (normalizer) and it was closely compared between HCV patients. For a second step in data analysis, we used comparative Ct method (ΔΔCt method) with the following formula: ΔCt = Ct (target, TLR) − Ct (normalizer, *β*-Actin). The comparative ΔΔCt calculation involved finding the difference between ΔCt of HCV patient and the mean value of the ΔCt from normals for each analyzed molecule. Fold increase in the expression of specific TLR mRNA in HCV patients compared to normal controls was also calculated as 2 − (ΔΔCt).

### 4.3. Statistical Analysis

Descriptive and analytical statistics were performed using the Excel 2010 software package (Microsoft Inc., Redmond, WA, USA). Data are presented as mean ± standard deviation (SD) and range. Categorical data are presented as numbers and percentages. Mean and median values were calculated. The *p* values ≤ 0.05 were considered statistically significant.

## 5. Results

### 5.1. Clinical Characteristics of HCV Patients

The majority of the study subjects were males (76%) followed by females at 24%. There was no significant difference in the mean ages among control and patient groups. The alanine aminotransferase (ALT) values of patients with relapsed HCV infection and in patients with liver cirrhosis were significantly higher with respect to the control values (*p* < 0.001). The baseline data along with the demographic characteristics are tabulated in Table 1. The median HCV RNA values of HCV infected patients with relapsed infection and patients who have progressed to cirrhosis of liver were significantly higher than those of the patients who were HCV infected but treatment naïve (*p* < 0.05). All the HCV patients were infected with HCV genotype 3, of which genotype 3a was the most predominant subtype (68.3%, *n*= 41) followed by subtype 3b (31.7%, *n*= 19) (data not shown).

### 5.2. TLR Gene Expression Analysis

**Figure 1:**
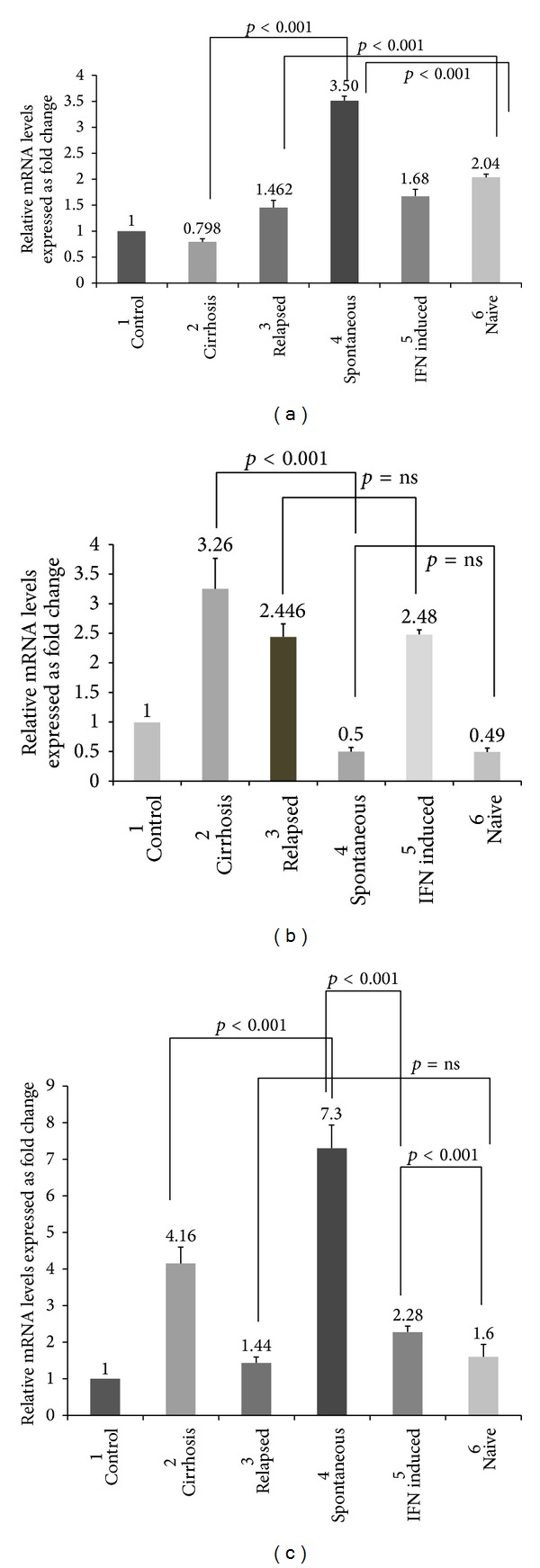
(a) Comparison of mRNA expression levels of TLR 3 expression within different stages of HCV infected population. *p* values ≤ 0.05 are considered to be statistically significant and ns: statistically nonsignificant. (b) Comparison of mRNA expression levels of TLR 7 expression within different stages of HCV infection population. *p* values ≤ 0.05 are considered to be statistically significant and ns: statistically nonsignificant. (c) Comparison of mRNA expression levels of TLR 8 expression within different stages of HCV infected population. *p* values ≤ 0.05 are considered to be statistically significant and ns: statistically nonsignificant.

Analyzing real-time PCR data by comparative ΔΔCt method indicated that expression of TLR 3 mRNA increased more than 3.5-fold in individuals who had spontaneously cleared the virus and twice in HCV infected individuals who have not received any IFN treatment compared with the healthy controls. Further analysis of the TLR 3 mRNA levels within different patient groups identified in the present study showed that treatment naïve HCV patients had significant increase in TLR 3 expression as compared to patients with relapsed infection (*p* < 0.001). Interestingly, people with HCV induced cirrhosis of liver showed more significant decrease in TLR 3 expression than individuals who had spontaneously cleared the viral infection (*p* < 0.001) (Figure 1(a)).

TLR 7 which is a sensor for viral single-stranded RNA and is believed to play an important role in viral hepatitis infection was upregulated 2.48-, 2.44-, and 3.26-fold more than the control values in IFN induced, relapsed, and cirrhosis patients, respectively. Decreased TLR 7 mRNA levels were found in patients who have spontaneously cleared the infection and in HCV patients who were yet to receive antiviral treatment (i.e., treatment naive). Comparison within different patient groups shows that TLR 7 mRNA expressions were not significantly upregulated in patients with relapsed infection and in individuals undergoing interferon treatment (*p* > 0.05, not significant), whereas results showed significant upregulation of TLR 7 mRNA in individuals who have progressed to liver cirrhosis stage compared to those who have spontaneously cleared the infection (*p* < 0.001) (Figure 1(b)).

TLR 8 which is also phylogenetically and structurally related to TLR 7 and can sense single-stranded RNA was analyzed. Results showed that TLR 8 mRNA levels were significantly upregulated (7.8-fold) with respect to control values. Additionally, patients with cirrhosis of liver reported 4.16-fold increases in mRNA levels with respect to control values. Univariate analysis within the patient groups shows that no significant fold change increase was noticed between HCV infected individuals with relapsed infection and in treatment naïve HCV patients. But TLR 8 mRNA expression levels were significantly increased in patients who were interferon treated versus those who were treatment naïve (Figure 1(c)).

## 6. Discussion

TLRs are sensors of the host innate immune system, which detects conserved molecular signatures of a wide range of microbial pathogens and initiates innate immune responses via distinct signalling pathways [20]. Intracellular localized TLRs 3, 7, 8, and 9 are of specific interest in patients with chronic viral infections since these receptors can recognize virus-derived molecular patterns [13–15]. TLR 3 senses double-stranded RNA representing viral infection [10]. A recent report by Li et al. (2005) indicates that HCV may use TLR 3 pathway to evade immune surveillance through HCV NS3/4A protease mediated cleavage of TLR 3 adaptor protein [21]. Molecular signals through TLR 3 ligands such as HCV dsRNA in its replicative forms enhance the receptor expression and responsiveness. Our results demonstrate that TLR 3 expression was upregulated in patients who had spontaneously cleared the infection and in HCV treatment naive patients, which suggest that TLR 3 mediates the establishment of the antiviral state against HCV infection (Figure 1(a)). Another interesting observation was that, along with TLR 3, TLR 7 expression was also elevated; it remains to be investigated further whether both TLR 3 and TLR 7 signalling mechanisms worked in tandem together to establish an antiviral state in HCV infected individuals.

HCV is a single-stranded RNA (ssRNA) virus; thus, it might act as a ligand for ssRNA specific TLR. But the specific role of TLR 7 and TLR 8 in innate immune response to HCV is yet to be understood. Viral recognition receptors, including TLR 7, activate IRF 7 upon recruitment of MyD88, a common TLR adaptor protein. Once the receptor is engaged, a complex is formed between MyD88, TRAF6, IRAK4, and IRAK1 that allows for activation of IRF [22, 23]. TLR 7 expression decreases with HCV replication; our results also support the statement; TLR expression in chronic HCV patients was found to be attenuated but, in patients with cirrhosis of the liver, the expression of TLR 7 was elevated. Therefore, elevated levels of TLR 7 were associated with progression of HCV infection (Figure 1(b)).

Interestingly, our findings showed that elevated levels of TLR 8, another key TLR which binds to ssRNA, were elevated in patients with cirrhosis and with relapsed HCV infection. Thus, we speculate that both TLR 7 and 8 are key molecules which are being upregulated in HCV infection (Figure 1(c)).

TLR 9 is activated by unmethylated CpG-rich bacterial and viral DNA acting through the myeloid differentiation primary response (MYD88) dependent signal transduction pathway [24]. Since HCV contains single-stranded RNA as its genetic material and does not produce any intermediate double-stranded DNA during its replication or transcription, we did not concentrate much on TLR 9. Also the role of TLRs 7 and 8 in HCV pathogenesis has not been studied extensively. Hence, we concentrated more on less studied TLRs 7 and 8 gene expression within our study samples. Initial study within our study population shows that TLR 9 mRNA expression levels were elevated in HCV patients with cirrhosis only, whereas in other groups the mRNA levels were not significantly elevated as compared to control population (data not shown). Thus, it can be said that within genotype 3 infected HCV populations TLRs 3, 7, and 8 are the key molecules which play a pivotal role in pathogenesis of HCV infection.

Our study encompassed a limited number of samples; evaluation of more samples shall improve the statistical validity of our results. Given their increasing importance in influencing host immunity, TLRs have the potential to serve as biomarkers for HCV pathogenesis; just as the C reactive protein is clinically used to assess HIV disease progression, monitoring the levels of specific TLRs could predict the rate of progression of HCV infection. To our knowledge, no other study has documented mRNA expression of TLRs 3/7/8 in HCV genotype 3 infected subjects within the different patient groups of Indian population. Ours is the first attempt of this kind from this region which analyzes TLR mRNA expression levels as an important host immune biomarker.

In conclusion, TLRs 3, 7, and 8 are prime biomarker candidates for HCV infection mRNA expression analysis which might improve current therapeutic approaches.

## Figures and Tables

**Table 1:** Baseline characteristics of patients enrolled in this study.

	Control	HCV infected (treatment naïve)	Recombinant IFN*α* treated	Spontaneous clearance	Relapsed	Cirrhosis of liver
Number of patients	*n* = 20	*n* = 20	*n* = 20	*n* = 20	*n* = 20	*n* = 20
Mean age (range), years	46 (30–62)	48 (31–64)	31 (31–54)	28 (20–48)	47 (30–60)	52 (33–68)
Gender (male : female)	10 : 10	15 : 05	14 : 06	14 : 06	18 : 02	15 : 05
Median BMI (range), kg/m^2^	26 (21–32)	24 (30–38)	26 (21–32)	30 (21–39)	25.5 (20–38)	26 (31–35)
ALT^!^ ± SD (IU/L)	37.42 ± 6.8	156 ± 6.3	121 ± 3.6	85 ± 5.8	126 ± 2.3	140 ± 8.5
AST^#^ ± SD (IU/L)	34.65 ± 14.22	118 ± 5.5	156 ± 3.0	114 ± .3	125.23 ± 2.6	156 ± 4.3
Median HCV RNA levels at baseline (10^3^ IU/ml)	—	425 (10–6500)	25 (25–650)	BDL	1560 (107–20000)	3265 (129–2000)

^!^Alanine aminotransferase level shown in IU/L with its standard deviations.

^#^Alanine aspartate aminotransferase level shown in IU/L with its standard deviations.

All enzymatic methods were done at 37°C, in a Beckman Coulter Synchron CX5 PRO, USA, instrument.

SD: standard deviation, BMI: basal metabolic index, and BDL: below detection limit.

**Table 2:** Primers used for SYBR Green PCR based amplification of TLRs in this study.

Toll receptors	Primers	Sequence	NCBI accession reference number	Start site	Stop site	Product size (bp)
TLR 3	Forward	5′-GTG CCA GAA ACT TCC CAT GT-3′	NM_003265.2	383	402	231
	Reverse	5′-TCC AGC TGA ACC TGA GTT CC-3′		594	613	

TLR 7	Forward	5′-AAT GTC ACA GCC GTC CCT AC-3′	NM_016562.3	782	801	223
	Reverse	5′-GCG CAT CAA AAG CAT TTA CA-3′		985	1004	

TLR 8	Forward	5′-TGT GAT GGT GGT GCT TCA AT-3′	XM_005274543.2	1028	1047	187
	Reverse	5′-ATG CCC CAG AGG CTA TTT CT-3′		1214	1195	

Binding site refers to the site where polymerase chain reaction (PCR) primer binds to prime duplication of a complement to the existing complementary DNA (cDNA) sequence of the mRNA; bp: base pair.

## Abbreviations

HCV: Hepatitis C virus
MAMPs: Microbe-associated molecular pattern
TLR: Toll like receptor
dsRNA: Double-stranded ribonucleic acid
ssRNA: Single-stranded ribonucleic acid
ALT: Alanine aminotransferase
AST: Aspartate aminotransferase
RT-PCR: Reverse transcriptase polymerase chain reaction
NCR: Noncoding region
MGB: Major groove binder
NIBSC: The National Institute for Biological Standards and Control
cDNA: Complementary deoxyribonucleic acid
SD: Standard deviation
IRF: Interferon regulatory factor
MyD88: Myeloid differentiation primary response gene
TRAF: TNF receptor-associated factors
IRAK: Interleukin-1 receptor-associated kinase.
